# Temporal changes of gene expression in health, schizophrenia, bipolar disorder, and major depressive disorder

**DOI:** 10.1038/s41537-024-00443-7

**Published:** 2024-02-17

**Authors:** Arsen Arakelyan, Susanna Avagyan, Aleksey Kurnosov, Tigran Mkrtchyan, Gohar Mkrtchyan, Roksana Zakharyan, Karine R. Mayilyan, Hans Binder

**Affiliations:** 1 Institute of Molecular Biology NAS RA, Yerevan, Armenia; 2 Armenian Bioinformatics Institute, Yerevan, Armenia; 3 Institute of Biomedicine and Pharmacy, Russian-Armenian University, Yerevan, Armenia; 4 Department of Therapeutics, Faculty of General Medicine, University of Traditional Medicine, Yerevan, Armenia; 5 Interdisciplinary Center for Bioinformatics, Leipzig University, Leipzig, Germany

**Keywords:** Molecular neuroscience, Schizophrenia, Target identification, Genetics of the nervous system

## Abstract

The molecular events underlying the development, manifestation, and course of schizophrenia, bipolar disorder, and major depressive disorder span from embryonic life to advanced age. However, little is known about the early dynamics of gene expression in these disorders due to their relatively late manifestation. To address this, we conducted a secondary analysis of post-mortem prefrontal cortex datasets using bioinformatics and machine learning techniques to identify differentially expressed gene modules associated with aging and the diseases, determine their time-perturbation points, and assess enrichment with expression quantitative trait loci (eQTL) genes. Our findings revealed early, mid, and late deregulation of expression of functional gene modules involved in neurodevelopment, plasticity, homeostasis, and immune response. This supports the hypothesis that multiple hits throughout life contribute to disease manifestation rather than a single early-life event. Moreover, the time-perturbed functional gene modules were associated with genetic loci affecting gene expression, highlighting the role of genetic factors in gene expression dynamics and the development of disease phenotypes. Our findings emphasize the importance of investigating time-dependent perturbations in gene expression before the age of onset in elucidating the molecular mechanisms of psychiatric disorders.

## Introduction

Mental disorders, such as schizophrenia (SCZ), bipolar disorder (BD), and major depressive disorder (MDD), are complex and heterogeneous conditions thought to arise from an interplay of diverse genetic and environmental factors^1^. Over the past decade, numerous studies have investigated the genetic and epigenetic changes that contribute to the development and progression of these disorders^2–5^. While it is clear that the molecular events contributing to disease manifestation and progression are distributed through embryonic to late stages of life^6–8^, relatively little is known about the temporal perturbations of the brain transcriptome that occur before onset and during each psychiatric disease. Because the age of onset of those neuropsychiatric disorders corresponds to adolescence and early adulthood, the clinical samples are always thresholded to the relatively older age. Thus, a direct comparison of clinical and control datasets is limited by the age of onset and does not include prenatal and early postnatal age ranges. This triggered multiple studies to understand the transcriptome dynamics across development^9–11^, and attempts have been made to overcome this issue by analyses of transcription of the disease-associated genes in pre- and postnatal control brains^11^ or by analyses of temporal changes in the samples with age-matched controls^10^. However, they are not free from limitations. Indeed, the first approach completely undermines the environmental factor-related epigenetic control. The second largely disregards the causative transcriptional perturbations during early neurodevelopment, contributing to a psychiatric disease later in life.

In the present study, we aimed to investigate the time-related changes in gene expression landscape and functional processes in the normal aging brain, schizophrenia, bipolar disorder, and major depressive disorder throughout the entire lifespan. First, we used a neural network-based self-organizing maps (SOM) machine learning and dimension reduction approach to transform multi-dimensional gene expression data into 2-dimensional transcriptome landscapes (or expression portraits) for each sample defined as a quadratic grid of microclusters of co-expressed genes called metagenes. Due to the self-organizing properties of the algorithm, metagenes with similar characteristics are located in adjacency on the grid, thus forming larger clusters of metagenes referred to as ‘spots’ or gene modules. Conversely, metagenes with differing profiles are located at distant grid locations. Notably, single genes are distributed over the metagenes of the portraits in an identical way, meaning that the portraits can be directly compared with one another^12,13^. As a part of the oposSOM R package^14^, the SOM method is complemented by comprehensive downstream analysis tools including visualization of individual samples and groups using expression heatmaps, differential expression analysis, diversity analysis, and biological function mining. oposSOM has been used in a large number of applications so far, such as transcriptomic studies of complex diseases including cancers^15–18^, aging^19,20^, and multi-omics integrative investigations^21,22^. Next, we used a Gaussian Process Regression approach^23,24^ to regress and impute the temporal perturbation points for the characteristic functional gene modules (or spots) in the brain transcriptome, contributing to these mental disorders from early postnatal life onwards. We also investigated the relationship of time-related transcriptome changes with brain cell population shifts and GWAS risk factors that may promote the development and progression of these disorders. Our results provide new insights into the contribution of different pathophysiological processes that may drive these conditions.

## Results

### Gene expression landscape in health, aging, and mental disorders

This study used eight publicly available datasets to analyze postmortem gene expression in the brain prefrontal cortex (PFC) of controls (CNTRL) and subjects with mental disorders (SCZ, BD, and MDD) generated using the Affymetrix Human Genome U133 Plus 2.0 microarray platform. Batch adjustment (for dataset batch, sex, pH, and post-mortem interval) considerably removed unwanted variability in gene expression data of the combined dataset, making it suitable for downstream analyses (Supplementary Fig. S1).

**Fig. 1:**
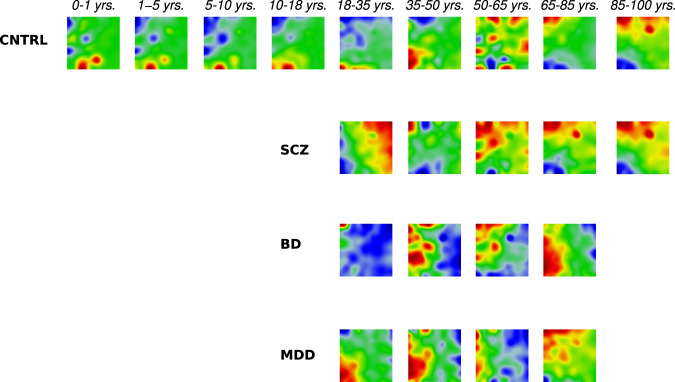
Group-specific (mean) transcriptome landscapes in control and mental disorder groups across age. The expression states of genes on portraits are represented in terms of color textures from blue to red, corresponding to under- and over-expression, respectively. The green areas on maps represent invariant genes. The same gene locations across portraits allow for a direct comparison. CNTRL Control (healthy brain), SCZ Schizophrenia, BD Bipolar Disorder, MDD Major Depressive Disorder.

Next, using the SOM method, we generated gene expression “portraits”^12,13^ across healthy and diseased (SCZ, BD, and MDD) samples spanning different age groups. These portraits visualize clusters of co-expressed genes in terms of gene (spot) modules within a “50 × 50” SOM grid image. Gene expression in each sample portrait is visualized using a color gradient, from blue to red, referring to under- and over-expression relative to the log-mean expression of each gene across all samples, respectively. It should be noted that the location of genes on SOM portraits is identical across all samples, which makes them directly comparable. For visualization purposes, we defined age category groups based on stages of normal brain PFC development and aging^25,26^. However, it should be emphasized that SOM training was performed in an unsupervised way, and age categories did not affect the localization of metagenes on the SOM grid and the clustering of genes on the transcriptome landscape^12,13^. Group-wise averaging of the expression portraits provides mean group portraits that highlight group-specific changes in gene expression landscapes (Fig. 1). For example, genes forming a spot on the left upper corner are downregulated (blue) in the CNTRL_(0-1] group, while the same gene spot is upregulated in controls after age 65 (Fig. 1).

**Fig. 2:**
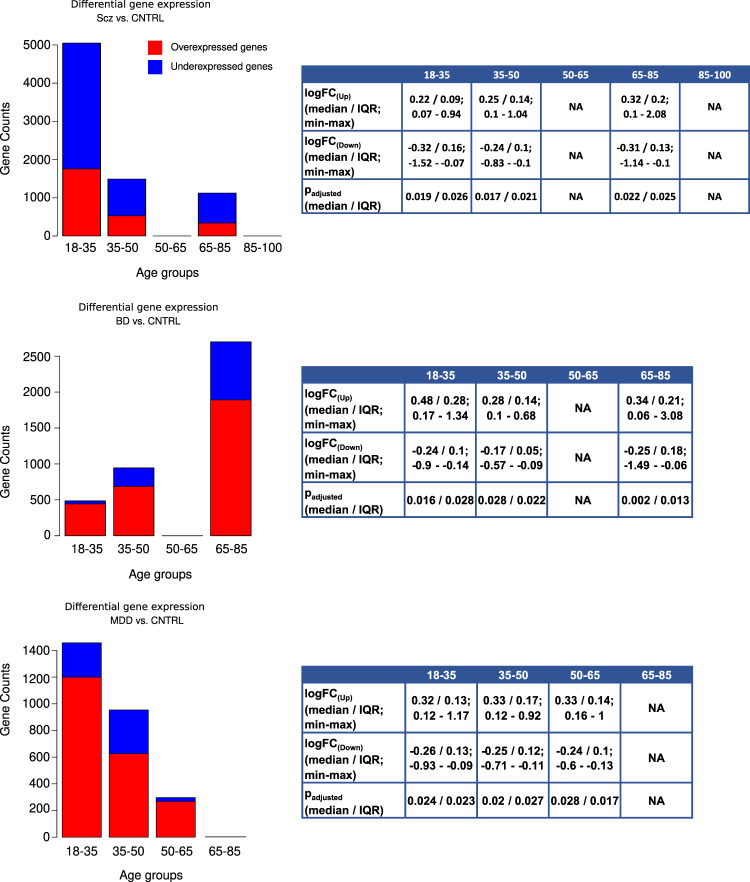
Distribution of differentially expressed genes across age groups in studied diseases. Red and blue bars indicate the number of over- and under-expressed genes in pairwise comparison of diseases and control samples of corresponding age groups. logFC ≠ 0 and FDR-adjusted *p* < 0.05 was used as a criterion for differential expression.

The results of gene expression profiling within nine age groups revealed temporal alterations, which were exclusive or shared by the transcriptome of the healthy and diseased brains. Concordant to our recent report, we observed transcriptional drifts of aging, suppressing the gene clusters activated before and activating the clusters that were “silent” at the early developmental stages^20^ (see Fig. 2 of corresponding publication). Particularly, SOM portraits demonstrate a complete shift in transcriptional activity in the control samples between 0-18 and 65+ years of age (Fig. 1).

It was also evident from SOM portraits that transcriptional abnormalities were differential across mental illnesses after the clinical manifestation of the diseases (Fig. 1). Compared to controls, the mean portraits for SCZ and BD in maturing (18-35 years) and early aging (35–50 years) brains showed considerably different distributions of high- and low-expressed gene modules. These differences became less pronounced in later aging stages (>50 years). Notably, the transcriptional landscape of SCZ patients aged between 50-65 years (Pearson r = 0.70, *p* < 2.2e−16) and 65-80 years (Pearson r = 0.84, *p* < 2.2e−16) closely resembled that of the senior controls (age >85). In contrast, the SOM portrait of BD at 50-65 years was considerably different from the corresponding control portrait (Pearson r = 0.17, *p* < 2.2s−16), while MDD (18-35 years) showed the closest profile to the young controls (Supplementary Fig. S2).

Further gene-wise analysis showed that schizophrenia and major depressive disorder were characterized by an age-dependent decline in the number of differentially expressed genes (DEGs). In contrast, differentially expressed gene counts in BD increased with age. On the other hand, we observed more downregulated DEGs in SCZ compared with BD and MDD (Fig. 2).

### Dissecting SOM transcriptome landscape into functional gene modules

**Fig. 3:**
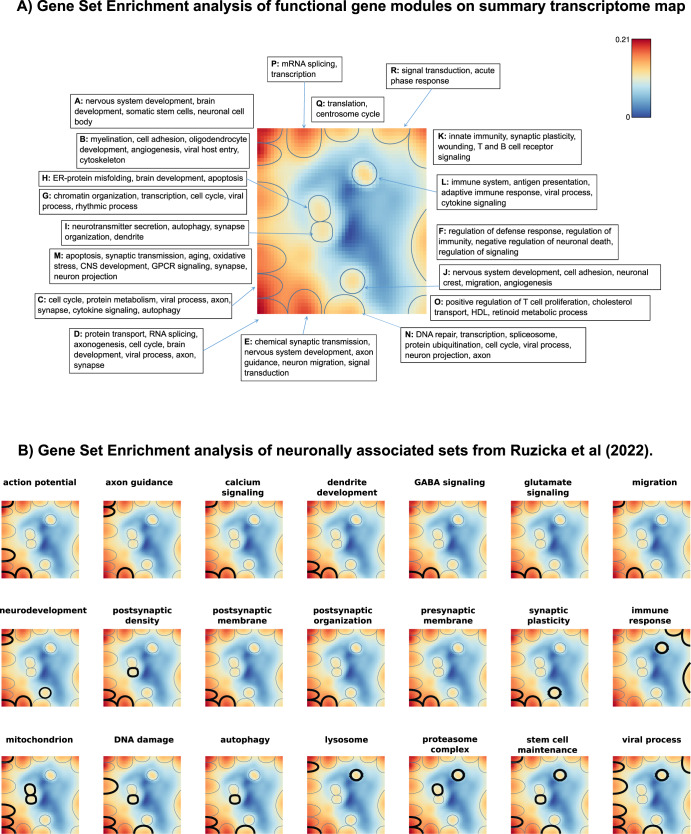
Functional dissection of transcriptome landscape. **A** The coloring gradient from blue to dark red indicates an increase in the variance of spot expression across all samples. Spots are indicated with blue lines, top Gene Ontology terms associated with spots presented in the corresponding boxes. **B** Mapping neuronally-associated gene sets onto the SOM spots (highlighted by bold black circles). It can be seen that the individual gene sets split across multiple spots. While in most instances these spots are closely positioned (as seen with dendrite development or presynaptic membrane gene sets), there are occasions when they appear on distant spots, such as with the “viral processes” gene set.

The entire gene expression landscape (global summary map) in the healthy and diseased aging brains was obtained by superposition of group SOM portraits^12^. It was then visualized in terms of gene expression variance, indicating regions (spots) of the highly variable and invariant genes, and, in addition, as an overexpression summary map where spots are visualized in terms of their expression values (Supplementary Fig. S3). The entire landscape was divided into 18 gene modules (spots) that were assigned to letters from “A” to “R” (Fig. 3A). Each spot is characterized in terms of the list of genes included and their mean expression profile in studied groups compared to the global average expression. This allows comparison of spots’ mean expression across groups (e.g., control vs. schizophrenia) as well as functional interpretation using enrichment or overrepresentation analysis methods^12–14^.

To decipher the biological functions of the genes in spots, we performed a gene set enrichment analysis using gene sets implemented in the oposSOM package as well as using curated 14 neuronally-associated functional categories from the previous report^27^ (Fig. 3B, Supplementary Table S1). Our results showed multiple spot associations with neuronal molecular functions, such as neurodevelopment, migration, calcium signaling, axon guidance, action potential, postsynaptic density, dendrite development, glutamate signaling, GABA signaling, postsynaptic organization, postsynaptic membrane, presynaptic membrane, and synaptic plasticity (Fig. 3A and B). These spots were located on the left part of the SOM portrait. In contrast, immune system-related spots were localized on the right side of the SOM variance map, which reflects anticorrelated gene activity compared with the neuronal functions. We also studied the association of cell senescence-related processes^28^ with spot expression. Mitochondrial function, DNA damage and repair processes, autophagy, lysosome, and proteasome genes also showed association with multiple spots and were co-localized with neuronally-associated spots. Stem cell maintenance was associated with spots A, C, I, L, and N. We also noticed multiple spot associations with the viral process (spots B, G, C, D, N, L), which reflect the activation of genes involved in RNA processing and immune system response. In terms of aging the healthy brain, the landscape suggests a consecutive activation/deactivation of neurodevelopmental, stemness, neuronal maturation, and inflammatory processes with increasing age (Fig. 1). These dynamics of healthy brain transcriptome allowed imputing time-perturbations of deregulated gene modules in the disease groups (see following subsections).

**Fig. 4:**
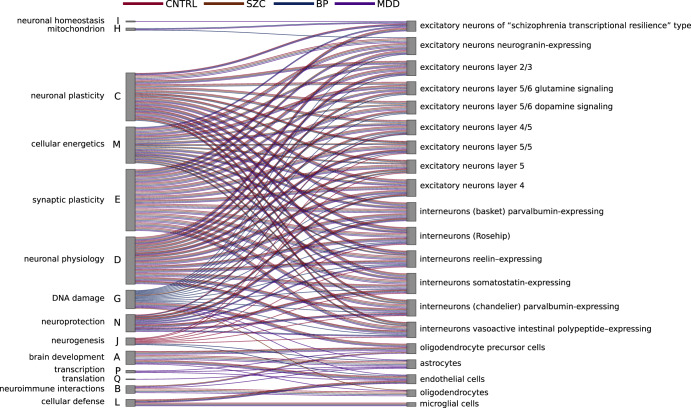
Association brain cell population gene markers with functional modules on transcriptome landscape. Pearson’s correlation coefficient r > 0.5 was selected as a threshold for visualizing the links between spots and cell signature Gene Set Z-scores.

Recently published single-cell (sc) RNA sequencing studies of brain regions and cell populations provided detailed insight into the composition of brain cell populations in health and disease (for review see^29^), as well as into their expression signatures^27,30^. Moreover, it has been demonstrated that changes in cell populations in the brain were associated with SCZ, BD, and MDD^31^. To correlate functional gene modules with the brain-specific cell populations, we calculated Gene Set Z-scores for brain cell gene sets from recent scRNA-seq publication^27^, and projected them on the generated SOM landscape (Fig. 4, Supplementary Fig. S4). Excitatory and inhibitory neuron populations were highly correlated with the expression of spots C, D, E, M, I, and G associated with neurodevelopment, axon and synapse development, neuron migration, signal transduction, neuron projection, and neurotransmitter secretion. The expression of glial cell signatures was correlated with spots A (neuronal stem cell population maintaining, cell adhesion - astrocytes, endothelium, oligodendrocyte precursor cells), L (inflammation, cellular and humoral immune response - microglia), B (myelination, oligodendrocyte development, cytokines - oligodendrocytes), G (cell cycle, growth factors - oligodendrocyte precursor cells), and P (cell metabolism, transcription, and translation - oligodendrocyte precursor cells).

### Temporal changes in gene expression in diseases and non-diseased brains

Next, we evaluated the age dependence of module expression in the healthy brain. The results indicate that neurodevelopment and neuronally-associated spots A, B, E, H, J, and M showed a strong negative association with age (Supplementary Fig. S5). We observed the loss of neuronal gene signatures and the increase of glial cell signatures with advancing age, in line with previous observations^3,20^ (Supplementary Fig. S6). In addition, we also noted several gene spots (F, G, I, K, O, R) that demonstrated increasing expression variance along the age axis without much changing the mean expression, which reflects higher transcriptomic volatility of the associated processes between the individuals. This increase of gene expression heterogeneity upon aging has been previously reported in several studies^32,33^ and was linked to the number of transcription factors that regulate expression on a single gene level^34^, somatic mutation accumulation^35^, and epigenetic alterations^36^. On the SOM transcriptome landscape, the spots with an increased gene expression variance but invariant mean expression were linked to regulation of cellular response (spots F, R, and P), neurotransmitter signaling and autophagy (spot I), immune response (spot K), cell cycle (spot G), and regulation of metabolism (spot O).

The expression of spots in corresponding age groups between the healthy and diseased brain samples was then compared using linear regression adjusted for age as a covariate. We observed significant upregulation of genes associated with neurogenesis (*p* = 0.004), cellular defense (*p* = 0.03), and translation (*p* = 0.00004) (spots A, L, and Q), and downregulation of neuronal plasticity (*p* = 0.0001), neuronal physiology (*p* = 0.00004), neuronal morphogenesis (*p* = 1e−6), cellular energetics (*p* = 0.03), and neuroprotection (*p* = 0.007) (spots C, D, E, M, N) in schizophrenia. In BD, the overexpression of neurogenesis (*p* = 0.0004), DNA damage (*p* = 0.05), and mitochondrion (*p* = 0.0008) (spots A, G, and H) were detected. Meanwhile, MDD was characterized by upregulation of DNA damage (*p* = 0.005), mitochondrion (*p* = 0.006), and cellular energetics (*p* = 0.015) (spots G, H, and M) and downregulation of immune response (*p* < 0.036) (spots F and K).

**Fig. 5:**
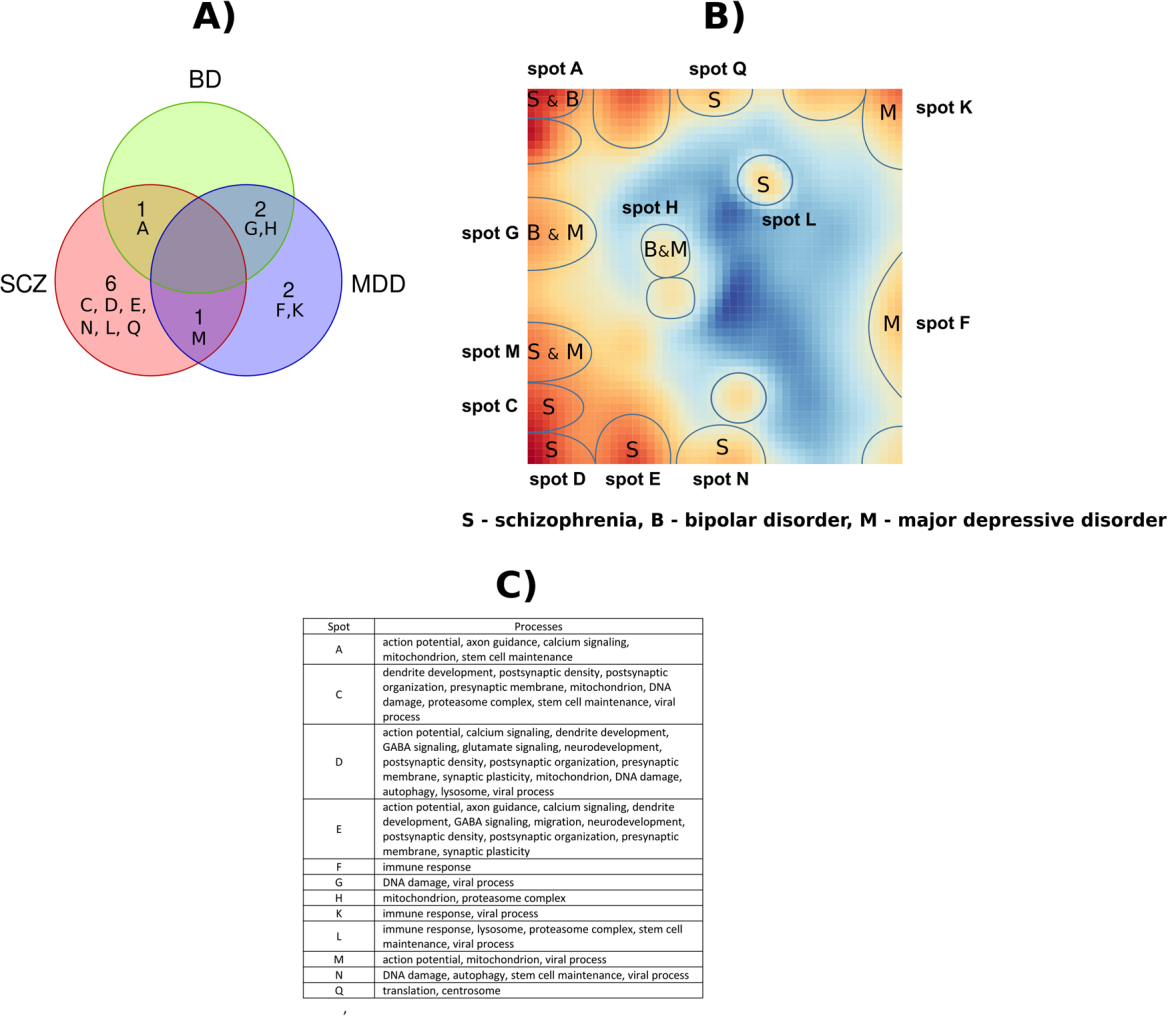
Differential expression of functional modules (spots) in SCZ, BD, and MDD. **A** overlap of the differentially expressed spots in diseases. **B** the SOM map with the respective modules associated with diseases. **C** functional annotation of differentially expressed modules.

We also observed an overlap between deregulated spots in disease groups (Fig. 5), in line with previous results on shared hereditary and underlying causative biology of mental disorders^37^ and similar to the results from Lanz et al.^38^.

Finally, we used the DEtime Gaussian Process Regression R package to predict the perturbation time of functional module expression between normal and diseased brains. The spot expression in SCZ, BD, and MDD as a function of age was compared to the expression of the normal brain in a pairwise manner. A likelihood ratio > 1 was used as a soft cut-off threshold to select time-perturbed differentially expressed spots^24^.

**Fig. 6:**
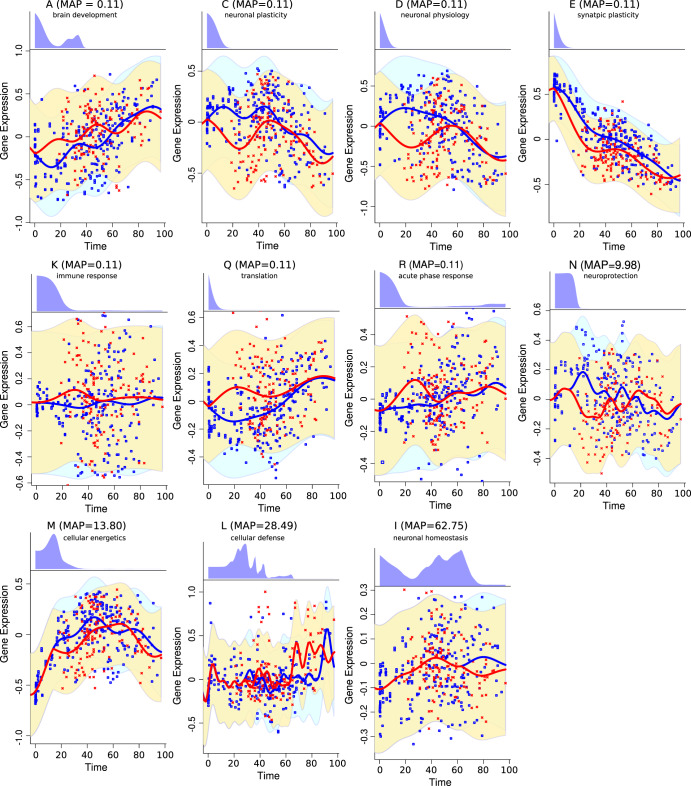
Time-dependent expression divergence of functional modules in schizophrenia. Red dots represent the spot expression in individual samples in the schizophrenia group, and blue dots represent the spot expression in the control group. The red and blue lines represent the Gaussian Regression Model fit for the diseased and control groups, respectively. The blue and yellow areas represent the 95% credible area for the disease and control regression models, respectively. The top panels for each spot show the inferred posterior distribution for the perturbation time.

Eleven out of 18 functional modules were time perturbed in schizophrenia (Fig. 6, Supplementary Table S2). Compared to controls, early gene expression perturbations with a maximum posterior (MAP) probability age of 0.11 years (40 days after birth) in schizophrenia include brain development, neural plasticity, neural physiology, synaptic plasticity, immune response, translation, and acute phase response (spots A, C, D, E, K, Q, R). On the other hand, the expression of functional gene modules associated with neuroprotection, cellular energetics, cellular defense, and neuronal homeostasis was characterized by late perturbations, covering adolescence to the elderly period (spots N, M, L, and I). The perturbation trajectories in schizophrenia suggested early downregulation of processes related to the maturation and refinement of the brain PFC circuits. Furthermore, the inspection of the perturbation profiles showed that the the expression of functional modules in schizophrenia and controls converge in the elderly (Fig. 6).

**Fig. 7:**
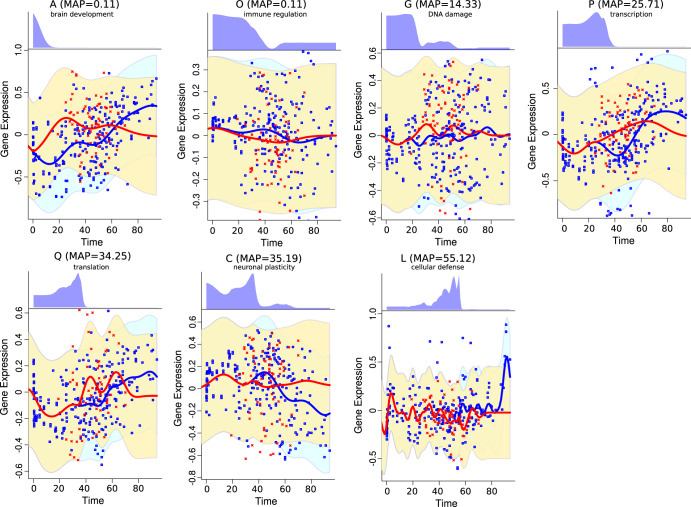
Time-dependent expression divergence of functional modules in bipolar disorder. Red dots represent the spot expression in individual samples in the bipolar disorder group, blue dots represent the spot expression in the control group. The red and blue lines represent the Gaussian Regression Model fit for the diseased and control groups, respectively. The blue and yellow areas represent the 95% credible area for the disease and control regression models, respectively. The top panels for each spot show the inferred posterior distribution for the perturbation time.

In BD early perturbations were observed in functional modules associated with brain development (upregulation) and immune regulation (downregulation), while late perturbations were associated with DNA damage and translation (upregulation) and downregulation of neuronal plasticity and cellular defense processes (Fig. 7, Supplementary Table S3).

**Fig. 8:**
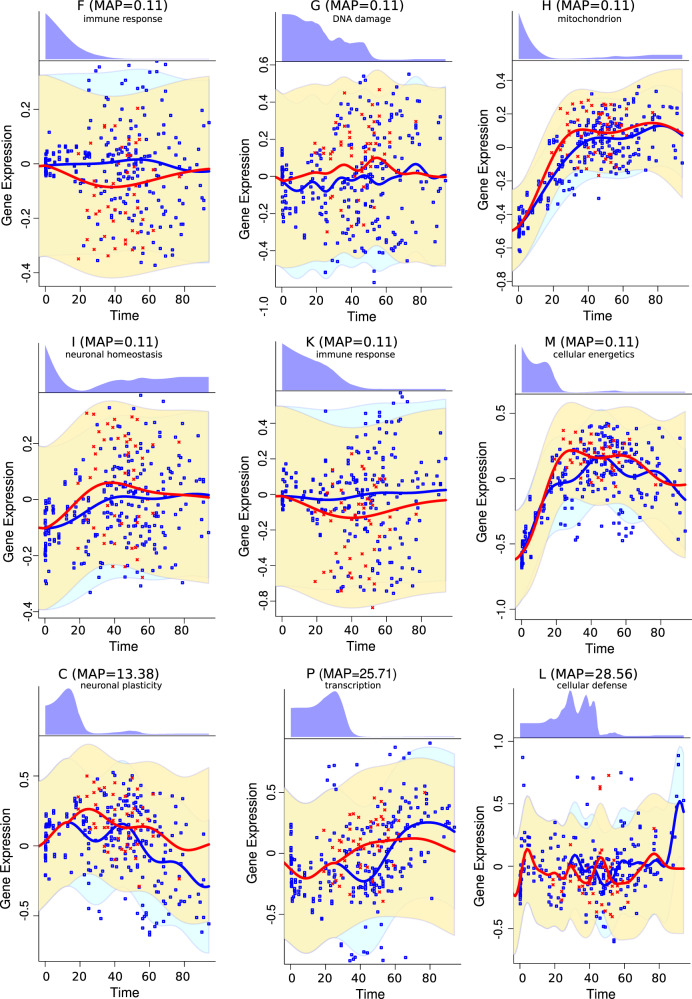
Time-dependent expression divergence of functional modules in major depressive disorder. Red dots represent the spot expression in individual samples in the major depressive disorder group, blue dots represent the spot expression in the control group. The red and blue lines represent the Gaussian Regression Model fit for the diseased and control groups, respectively. The blue and yellow areas represent the 95% credible area for the disease and control regression models, respectively. The top panels for each spot show the inferred posterior distribution for the perturbation time.

In MDD compared to controls, we observed early downregulation of immune response and DNA damage and upregulation of mitochondria-related genes, cellular energetics, and neuronal homeostasis. The late perturbations in the diseases included upregulation of neuronal plasticity, transcription, and downregulation of cellular defense/immune response (Fig. 8, Supplementary Table S4).

**Fig. 9:**
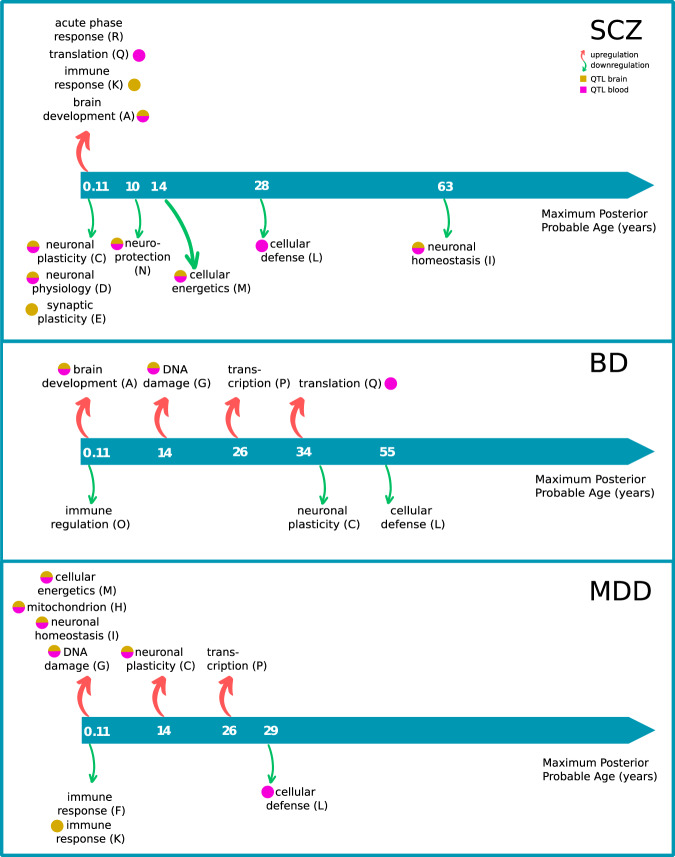
The simplified timeline of perturbations of gene functional modules in mental disorders. One can see that despite the overlap of the perturbed modules in these diseases, in the majority of cases, they are not consistent for the age period and perturbation trajectories. Additionally, most of the time-perturbed spots were enriched with eQTL signal-containing genes for blood and brain (adjusted Fisher Exact *p* value < 0.05; see Supplementary Fig. S7).

We also noticed an overlap in time-perturbed biological processes and functional modules (9 out of 18) between studied diseases (Fig. 9, Supplementary Table S5). However, only four overlapping spot perturbations were consistent for the age period, and only two of those had similar perturbation trajectories. Particularly, SCZ and BD shared overexpression of the brain development-associated genes (spot A) deregulated at age 0.11 years, and BD and MDD had similar perturbation patterns of transcription (spot P) at ~ 26 years of age.

Previously established associations of functional gene modules with brain cell gene signatures made it possible to describe observed changes in terms of brain cell populations. The time-perturbed differential expression showed that in schizophrenia, there were two events of neuronal cell signature loss. At 40 days after birth, there was downregulation of the gene signatures of both excitatory and inhibitory neurons, while at a late age, only the excitatory neuron signature was downregulated. In bipolar disorder, we didn’t observe early changes in the expression of neuronal signatures. Instead, late upregulation of both excitatory and inhibitory neuronal signatures was observed. Particularly, the neuronal cell signature perturbations were observed at 14 (spot G upregulation) and 35 years of age (spot C with dynamic down then up fluctuation from the norm), which involved almost all types of excitatory and inhibitory neurons. Both SCZ and BD showed marked early perturbation of glial signatures, especially related to astrocytes, and late perturbations associated with microglia. MDD was characterized by early upregulation of excitatory neuronal signature and downregulation of inhibitory signatures, while later in age, both signatures were upregulated.

### QTL enrichment of functional gene modules

The contribution of genetic factors in the development of schizophrenia, bipolar disorder, and major depressive disorder is well documented in many studies^39–41^. However, functional links between identified genetic loci and disease mechanisms have not been extensively explored. Here we explored whether time-perturbed functional modules on the SOM map were enriched with expression quantitative trait loci (cis eQTL) genes across 47 GTEx tissues. The majority of all time-perturbed spots were also enriched with eQTL signal-containing genes for blood and brain as well as other tissues (Fig. 9, Supplementary Fig. S7). Furthermore, the spots enriched with brain cell signatures (A, C, B, G, H, I, N) also showed enrichment for brain cortex eQTLs. Interestingly, brain cortex and blood eQTLs were enriched in the same spots, in line with the comparability of gene expression signatures in the brain and blood^42–44^. In addition, whole blood eQTLs were enriched in spots (D - Fc-epsilon receptor signaling, L - celular defense/immune response, and Q - translation).

Finally, we extracted eQTL genes from spots and performed gene set analysis against known GWAS resources (GWAS Catalog, DisGenNet, UK biobank), and others using the Enrichr and DOSE gene set analysis tool^45–47^. The analysis demonstrated that eQTL genes were also significantly associated with SCZ, BD, and MDD (p_adj_ < 0.05, see the term associations in Supplementary Table S6).

## Discussion

In this study, we used a combination of SOM and Gaussian Process Regression approaches to characterize time-related changes in gene expression and functional processes in the normal aging brain and mental disorders and to infer the temporal windows of their initial divergence. Our SOM analysis approach allowed for transforming the complexity of the expression of tens of thousands of genes into several dozens of co-expressed functional gene modules. This facilitated the association of these modules with diseases, as well as enabled the exploration of their biological functions. Using Gaussian Process Regression we were able to infer the temporal aspects of perturbations in these modules in comparison with healthy brain postnatal development, maturation and aging.

Our results showed that the entire transcriptome landscape in patients could be decomposed into partially overlapping age and disease-associated functional gene modules (spots).

First, using a larger set of control samples spanning a wider postnatal age range, we replicated our previous finding showing the dynamic changes in neurodevelopment, synaptic plasticity, and decline of neuronal function during lifespan^20^. Particularly, aging was paralleled with the increase of glial cell signatures and the decline of brain neuronal cell signatures indicating loss of neurons^48^. These results on temporal dynamics of gene expression in the human prefrontal cortex also correspond to the changes in DNA methylation levels reported previously and, in part, can be linked to the epigenetic alterations across the postnatal lifespan^3^. Particularly, the widespread methylome changes in the prefrontal cortex that occur during the transition from fetal into postnatal life appear to track first the loss of immature neurons before birth, followed by the rise of non-neuronal cell types through adulthood^3^.

The transcriptome landscapes in schizophrenia, bipolar disorder, and major depressive disorder showed considerable parallels with the aging brain. However, those were shifted toward younger age in patients in terms of biological processes such as loss of neurons^49,50^, DNA methylation^3,51,52^, immune response^53–56^, and loss of cognitive function^57,58^. Thus, our results support the concept of accelerated brain aging in SCZ, BD, and MDD^59^. However, the timing of gene expression perturbations suggests the primary role of neurodevelopmental aberrations in the development of those psychiatric disorders^6–8^.

We also observed an overlap in deregulated functional gene modules/processes between schizophrenia, bipolar disorder, and major depressive disorder. Both schizophrenia and bipolar disorder were characterized by the up-regulation of functional gene sets associated with astrocyte function, such as myelination, and brain development^60^. BD and MDD shared deregulation in oxidative stress^61,62^, hypoxia^63^, and oligodendrocyte function^64,65^. On the other hand, schizophrenia and MDD shared deregulation in gene modules for ER-protein misfolding, cell cycle, and other processes associated with excitatory and inhibitory neurons^66,67^. However, the direction of the changes differed across studied conditions in line with the previous results^68^.

Another worth noting observation is that there is a gradual decline in the number of differentially expressed genes in SCZ and MDD with aging. Our results correspond with the model developed by Demro et al. (2022) that showed no difference among people with psychiatric illnesses or their relatives compared to controls in advanced age^69^. The impaired cognitive and general functioning associated with advanced age in combined samples was not observed, which again emphasizes the major contribution of neurodevelopment-driven structural brain abnormalities^69^. In line with these findings, our results prompt the question of whether mental disorder studies in advanced age groups are feasible, and emphasize the importance of investigating time-dependent perturbations in gene expression. While the lower age limit in diseased groups in this study was similar to other reports, we used the Gaussian Process Regression^24^ to study the temporal perturbations of functional gene modules in the brain transcriptome of schizophrenia, bipolar disorder, and major depressive disorder prior to the age of onset.

Inferring the time-perturbation from transcriptome data showed that mental disorders are characterized by early, mid-, and late deregulation of identified functional spots. The early perturbations in schizophrenia were linked to neurodevelopment and synaptic plasticity, while late events included a marked fluctuation in microglial and immune markers in the brain^70^. In bipolar disorder, the early events were associated with T cells, which are known to play a crucial role in the fetal-to-adult brain transition^71^, as well as the elevation of astrocyte markers, while the late events were associated with neurodegeneration and neuroimmune modulation. The early events in MDD were mostly associated with inhibitory neurons, consistent with the previous results^72,73^.

Overall, our results align with the hypothesis that two or more ‘hits’ are required over the lifespan rather than only one early-life event for disease manifestation^74^. Maynard et al. (2001) reported that the cell-cell signaling pathways involved in nonaxial induction, morphogenesis, and differentiation in the brain could be considered a “first hit”, while destruction of the neuronal network might serve as a “second hit”^75^. Moreover, these observations are in agreement with previous studies indicating the involvement of both early and later neurodevelopmental alterations in the etiology and pathogenesis of schizophrenia^76^.

Time-perturbation analysis brought several worth-noting observations about the association of immune response with studied diseases. In the four gene modules enriched for immune processes (i.e., spots F, K, L, and R, see Supplementary Table S5), transcriptome perturbations occurred in the very early days of postnatal life except in module L. The gene clusters F and R, unique for MDD and SCZ, respectively, were enriched for biological processes such as regulation of immune response, cellular defense response, negative regulation of neuronal death, positive regulation of transcription by RNA polymerase II, negative regulation of TNF production and signal transduction. The downregulation of the gene module K enriched for innate immune response, negative regulation of cell proliferation, and signal transduction precede MDD, while its upregulation in the same period is observed for SCZ. Thus, none of those transcriptional perturbations can serve as hallmarks of neuroinflammation and aging, but rather as a result of the genetic/epigenetic response to early developmental adversities^77,78^. In all three psychiatric illnesses, the only late and incoherently complex perturbations were observed by the gene module L with distinct cellular indexing to the components of the blood-brain barrier (BBB), i.e. endothelial cells and microglia^79^․ Thus, the effect of medication^80^ and poor hygiene^81^ should also be considered together with the genetic load for perturbations in this immune cluster. Remarkably, during the initial deregulation periods, the BBB cellular components were decreasing relative to the norm in all three mental disorders (Supplementary Table S5). In schizophrenia, these data corroborate the recent in vivo findings of altered function or lower density of brain immune cells in the frontal cortex of the patients^82^. However, the upregulation of this cluster in SCZ after age 65 is likely to be secondary to the disease pathophysiology, treatment, and other environmental factors and probably can not contribute to the disease causative biology^83^.

Our results also indicate that the time-perturbed functional spots were enriched with eQTL genes, implying the contribution of genetic variance in developing a disease phenotype. Moreover, our gene set enrichment analysis showed that a significant portion of these eQTL genes was also associated with the genetic risk of developing studied diseases. Indeed, numerous studies reported the association between common and rare genetic variants with schizophrenia^84^, bipolar disorder^85^, and major depressive disorder^86^. Thus, the early perturbations could suggest the contribution of genetic components, while late perturbations could be more attributed to the effect of environmental factors and maturation. The previous study based on neurodevelopmental transcriptome and genotype association data showed early events strongly associated with schizophrenia than with bipolar disorder^11^. The same results have been observed for schizophrenia and MDD^87^. It is also notable that there is an overlap in functional modules between all three disorders, again emphasizing overlaps in molecular mechanisms and genetic associations^88^.

Collectively, our results indicate that psychiatric disorders are associated with the deregulation of multiple biological processes covering various aspects of cellular physiology and functions in the brain that are distributed over time. Our findings align with outcomes from other co-expression cluster identification studies. For instance, Gandal et al. pinpointed roughly 400 uniquely enriched gene sets (both GO and KEGG) within 13 co-expression modules shared across autism, schizophrenia, bipolar disorder, depression, and alcoholism (as detailed in Supplementary Data Table S2 of the cited reference)^88^. Likewise, Kang et al.‘s research identified approximately 370 unique enriched gene sets across 29 WGCNA clusters, detailing the transcriptomic changes in different brain regions throughout aging (refer to Supplementary Table 9 of the cited reference)^89^.

Several limitations of this study have to be considered. Firstly, we only included post-mortem samples from the prefrontal cortex. It is well documented that the different brain regions are characterized by variability of gene expression perturbations^90^. For example, a study showed considerable differences in gene expression and associated pathways in the striatum compared to the prefrontal cortex in patients with SCZ, BD, and MDD^38^. However, transcriptome changes in PFC can provide an important insight into the development of mental diseases as it is considered the control hub of the brain and involved in diverse functions related to information processing, memory, cognitive and emotional processes^91^.

Secondly, we did not consider the temporal changes in blood, though the involvement of systemic immunity/inflammation is evident in these diseases^92^. It could be beneficial to include a different “control” disease in our dataset to control downstream “non-causative” biological processes such as oxidative stress and inflammation. Nevertheless, by analyzing temporal profiles, we were able to map perturbations in biological processes over time thus differentiating between early (possible “driver”) and late (possible “passenger”) processes.

Third, we used microarray data in our analysis. While there are RNA sequencing datasets available for schizophrenia, BD, and MDD, to our knowledge, there were no publicly available datasets that could be combined to form a dataset with demographic characteristics comparable to our integrated dataset. On the other hand, to get comprehensive functional insight from bulk microarray data, we incorporated the information on gene sets associated with biological processes and cell populations from scRNA sequencing studies into our analyses. scRNA sequencing has inherent limitations on the number of samples to be analyzed, and combining it with the multiple sample processing capacity of microarray can strengthen the analysis.

Fourth, we acknowledge that some vital information was missing for patients, such as medication, disease duration, etc. Including this information would improve our model for temporal changes in gene expression.

Despite the mentioned limitations, our machine learning approach was able to trace normal aging and mentally ill brain transcriptome dynamics specific for each disorder, and distinguish genetic, functional, cellular and temporal characteristics. This study demonstrated that there are several transcriptome and functional process perturbations in mental disorders across the lifetime, which supports the multi-hit hypotheses of the disease development and progression. Moreover, our data indicate that early perturbation events can be more associated with genetic variation, while environmental factors can drive later events. Particularly, the causative transcriptional perturbations during early neurodevelopment largely contribute to schizophrenia and major depressive disorder later in life, while for bipolar disorder, transcriptional perturbations from the norm prevail in adolescence and early adulthood, emphasizing a major role of the relatively later interactions between causative genes and environmental factors in this disorder. Lastly, our results underline the age limit (<65 years) that should be applied for brain transcriptome studies of psychiatric diseases of neurodevelopmental origin, as, later in life, the brain transcriptome landscape of mental illnesses tends to unify to that of the normal aging brain profile.

## Materials and Methods

### Datasets

We used six datasets from Gene Expression Omnibus (GSE21138, GSE53987, GSE92538, GSE17612, GSE11512, GSE13564)^93^ and two datasets from The Stanley Medical Research Institute Online Genomics Database (Chen_study4, Dobrin_study5)^94^. GSE11512 and GSE13564 datasets contained gene expression profiles from post-mortem prefrontal cortex tissue from healthy individuals of varying ages 0-81 years. The GSE21138, GSE53987, GSE92538, and GSE17612 contained gene expression profiles of post-mortem brain samples from patients with schizophrenia (SCZ), bipolar disorder (BD), major depressive disorder (MDD), and matching controls ranging from 18 to 81 years. Chen_study4 and Dobrin_study5 contained gene expression profiles in the prefrontal cortex from mental disorder cases and matching controls.

Common phenotypic characteristics of samples across all datasets included primary diagnosis, age, sex, post-mortem interval in hours, and pH of the post-mortem brain tissue.

Gene expression in all datasets was measured with Affymetrix Human Genome U133 Plus 2.0 arrays (GEO Platform GLP570). The platform contains 54675 probes spanning the majority of protein-coding genes and transcripts.

### Data preprocessing, harmonization, and age grouping

Raw Affymetrix CEL files have been downloaded for all the datasets. Probe signal intensity conversion, background correction, RMA normalization, and log transformation were performed using the “affy” R package^95^. The resulting gene expression matrices were harmonized using RemoveBatchEffects from the “limma” R package^96^, controlling for dataset batch, brain pH, post-mortem interval, and sex. The resulting dataset was normalized by subtracting the log expression value for a gene from the mean value of that gene across all samples, which resulted in obtaining log fold change values relative to the average gene expression. Samples were assigned to age groups according to the following ranges based on stages of normal brain PFC development and aging^25,26^:

(0-1], (1-5], (5-10], (10-18], (18-35], (35-50], (50-65], (65-85], (85-100] years (Table 1).

**Table 1:** The number of samples in studied groups by age.

Age	Control	SCZ	BD	MDD
(0-1]	26	–	–	–
(1-5]	12	–	–	–
(5-10]	15	–	–	–
(10-18]	37	–	–	–
(18-35]	59	25	16	14
(35-50]	6	59	34	18
(50-65]	56	25	21	13
(65-85]	33	33	2	1
(85-100]	9	3	–	–
Total	253	145	73	46

### Clustering, identification of co-expressed modules, and functional analyses

The analysis of gene expression was performed with the “oposSOM” self-organizing maps (SOM)-based transcriptomic pipeline described in detail elsewhere^12,14^. Briefly, SOM training transforms the N (genes) x M (samples) gene expression matrix into a K x M metagene matrix, where metagenes represent the clusters of genes with a similar expression profile across all samples. The SOM algorithm initializes a k x k = K grid of nodes (metagenes) each described with a 1 x M weight vector. During the training phase, SOM clusters genes into metagenes based on the similarity between metagene’s weight vector and gene profile vector (the expression of a gene across the samples in the dataset) using the Euclidean distance metric. Thus, gene locations in the SOM grid are the same for all samples. Because of the linear initialization of SOM weight vectors, the profiles of adjacent metagenes are more similar to each other than to the distant ones. Consequently, if weights for a single sample are selected from all metagenes’ weight vectors they will form a K x 1 vector that represents the state of metagene expression for a given sample (sample SOM heatmap portrait). The metagene values for each sample can be visualized on a two-dimensional k x k grid by using a color code for expression values (from blue to red for underexpressed and overexpressed metagene values, respectively), which highlights clusters of up- and down-regulated genes from neighboring metagenes collectively referred to as spots or modules^13^. Sample-specific spots are then transferred to the global expression or variance summary map that allows direct sample- or group comparisons. The oposSOM analysis algorithm offers several criteria to select differentially expressed gene modules (spots) from the global summary map^14^, which we used to dissect our transcription landscape into functional gene modules.

Functional analysis of spots was performed using the Fisher’s Exact test and Gene Set Z-score against a gene set collection available in the “oposSOM” package^14^ as well as additional gene sets from a recent single-cell RNA sequencing publication^27^. REVIGO was used to simplify lists by grouping GO terms with similar functions^97^.

### Regressing time-dependent changes in modules in health and disease

Inference of perturbation time in gene modules on the SOM transcriptome landscape was performed using the DEtime R package^24^. Briefly, it is a Bayesian approach that uses non-parametric Gaussian Process Regression (GRP) to detect the time points of gene expression perturbations between controls and disease groups. GPR sets a Gaussian Process Prior over potential functions and then updates those based on observed data to obtain a posterior distribution. GPR is particularly powerful due to its inherent flexibility, allowing it to model intricate, non-linear associations without the necessity of a stipulated functional form. Moreover, compared to spline interpolation, GRP uses a full probabilistic model, while splines generally provide only point estimates and require prior knowledge about the number, and location of knots. In our analysis, instead of genes we used SOM modules (see above) to derive the time when the expression of the module changed in a disease compared to the controls.

### QTL enrichment

The functional modules showing differential association with age in controls and diseases were further evaluated for QTL enrichment in different tissues. Genotype-Tissue Expression (GTEx)^98^ single-tissue cis-eQTL dataset (v8) was used as a source. The enrichment of genes with QTLs in spots was evaluated in 47 tissues using a one-tailed Fisher exact test. Bonferroni correction was applied to Fisher’s *p*-values for each spot-tissue comparison.

### Supplementary information

Supplementary Fig.s and Tables

## Data Availability

The data associated with this study is deposited in the Zenodo open repository (https://zenodo.org/record/7936218/). The code for reproducing the results of this study is available in the GitHub repository (https://github.com/susieavagyan/BrainExp-TemporalDynamics).
