# Supplementary material for: Temporal changes of gene expression in health, schizophrenia, bipolar disorder, and major depressive disorder
Source: Schizophrenia (Heidelb). 2024 Feb 17;10(1):19. doi: 10.1038/s41537-024-00443-7 (PMC10874418; doi:10.1038/s41537-024-00443-7)
Supplement: Supplementary file 1 — Supplementary Fig.s and Tables [file 41537_2024_443_MOESM1_ESM.docx]

## Temporal changes of gene expression in health, schizophrenia, bipolar disorder, and major depressive disorder

## Supplementary Data


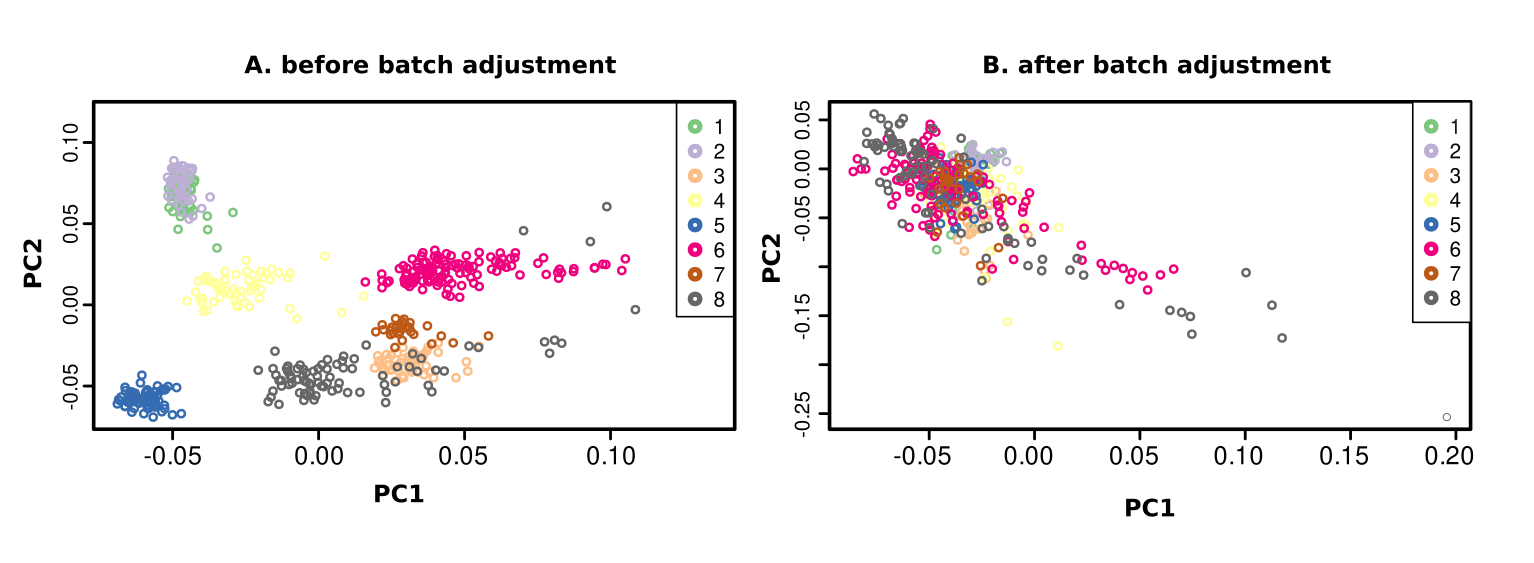


**Figure S1.** Batch adjustment and data normalization for downstream analysis. Colors indicate the dataset batches used in this study. A) Before adjustment, distinct clusters representing samples from each dataset are seen, indicating the presence of the strong batch effect. B) Application of the RemoveBatchEffects function from the limma R package considerably corrected the dataset-associated batch effects (see Methods Section of the main manuscript).


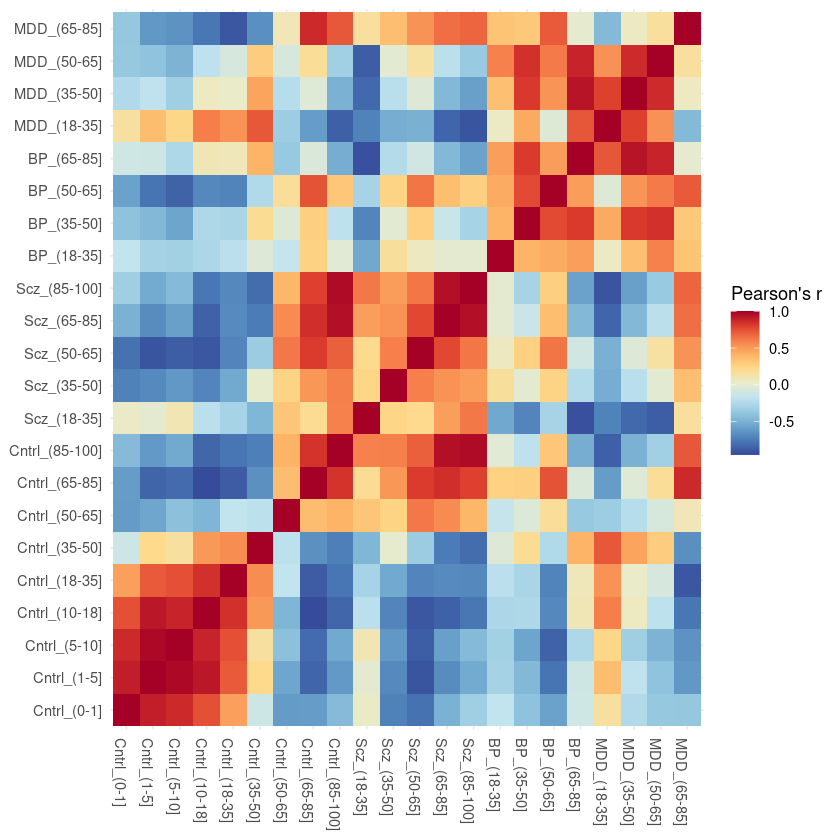


**Figure S2.** Pairwise correlation heatmap of group SOM portraits of age categories in schizophrenia, bipolar disorder, and major depressive disorder. The heatmap visually represents the Pearson correlation coefficients between various age categories in disease cohorts.


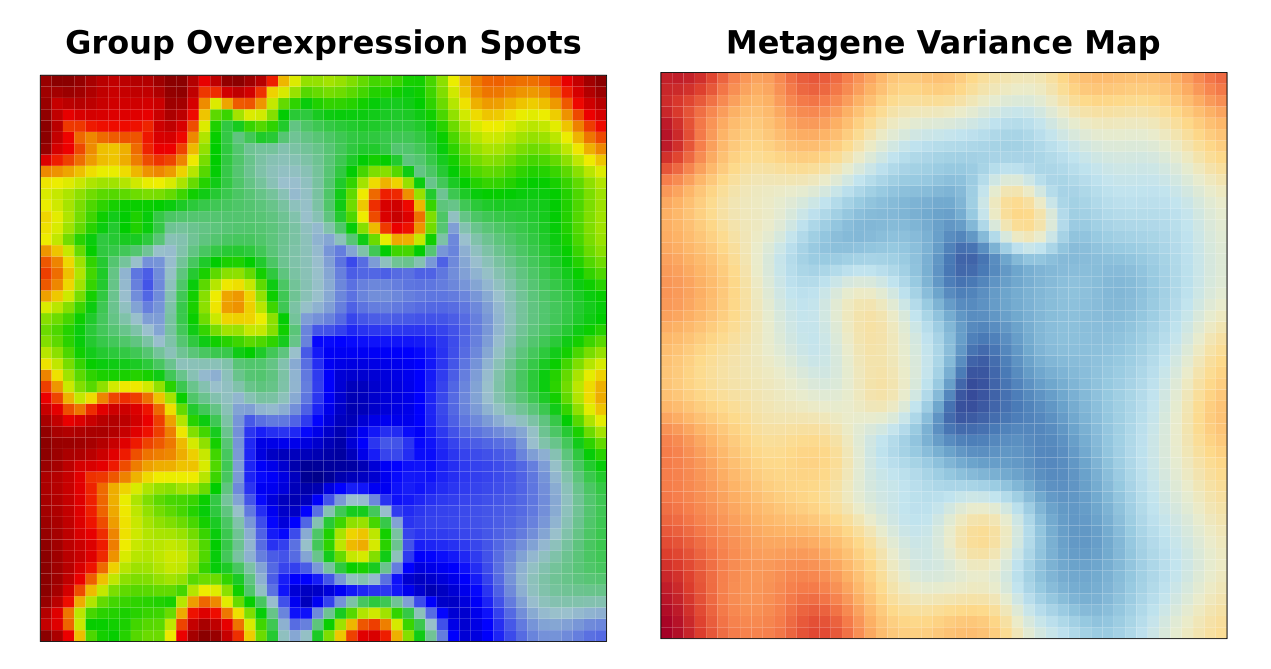


**Figure S3.** Global expression and variance SOM maps. The entire gene expression landscape in the healthy and diseased aging brains was visualized in terms of their expression values (blue to red colors corresponding to low-high expression, respectively) and gene expression variance, indicating regions of the high variability of gene expression (maroon) and invariant genes (blue).

**Table S1.** Association of curated neuronally-associated functional gene sets [(Ruzicka et al. 2020)](https://www.zotero.org/google-docs/?gb9s4J) with gene spots on SOM global transcriptome landscape of mental disorders and controls.

| Process | Spot | Top 3 correlated genes in the spot |
| --- | --- | --- |
| action potential | A | NTRK2(r=0.91); SLC1A3(r=0.897); ATP1A2(r=0.895) |
|  | D | PAFAH1B1(r=0.914); PRNP(r=0.905); FGF12(r=0.898) |
|  | E | NETO2(r=0.93); NETO2(r=0.87); KCNV1(r=0.85) |
|  | M | SCN2B(r=0.867); KCNQ5(r=0.842); CAB39(r=0.832) |
| axon guidance | A | BMPR1B(r=0.923); RHOBTB3(r=0.903); PALLD(r=0.882) |
|  | B | CNTN2(r=0.894); NCAM1(r=0.892); GAB1(r=0.874) |
|  | E | EPHA5(r=0.88); PTPRO(r=0.874); B3GNT2(r=0.859) |
| calcium signaling | A | ATP1A2(r=0.895); GJA1(r=0.894); FGF2(r=0.892) |
|  | D | DNM1L(r=0.915); PRNP(r=0.905); VDAC1(r=0.899) |
|  | E | CASK(r=0.89); GRIN2A(r=0.843); GRIN3A(r=0.828) |
| dendrite development | C | HPRT1(r=0.903); PAFAH1B1(r=0.893); CAPRIN1(r=0.883) |
|  | D | DNM1L(r=0.915); PAFAH1B1(r=0.914); ARF4(r=0.9) |
|  | E | GRIN3A(r=0.828); CDKL5(r=0.819); MARK1(r=0.819) |
| GABA signaling | D | GABRG2(r=0.878); USP46(r=0.859); GABRA1(r=0.849) |
|  | E | CNR1(r=0.798); TAC1(r=0.757); NLGN1(r=0.742) |
| glutamate signaling | D | PRNP(r=0.905); NRXN1(r=0.864); DLG1(r=0.856) |
|  | E | NETO2(r=0.93); SSTR1(r=0.884); NETO2(r=0.87) |
| migration | A | NTRK2(r=0.91); EMX2(r=0.884); AXL(r=0.861) |
|  | E | FGF13(r=0.839); CDKL5(r=0.819); MARK1(r=0.819) |
| neurodevelopment | A | NTRK2(r=0.91); EMX2(r=0.884); AGT(r=0.88) |
|  | B | HHIP(r=0.911); GPRC5B(r=0.904); ASPA(r=0.899) |
|  | D | DNM1L(r=0.915); PAFAH1B1(r=0.914); MYT1L(r=0.906) |
|  | E | PLPPR5(r=0.911); FGF13(r=0.839); MMD(r=0.834) |
|  | J | SOX11(r=0.882); DPYSL3(r=0.838); SOX11(r=0.828) |
| postsynaptic density | C | RHEB(r=0.888); NPTN(r=0.879); DNAJC6(r=0.869) |
|  | D | PRNP(r=0.905); KPNA1(r=0.879); MAPK1(r=0.87) |
|  | E | NETO2(r=0.93); NECTIN3(r=0.897); PLPPR4(r=0.893) |
|  | I | PSD(r=0.77); MAPK8IP2(r=0.749); PRRT2(r=0.712) |
| postsynaptic membrane | C | GLRB(r=0.89); NPTN(r=0.879); CNKSR2(r=0.865) |
|  | D | CNTN1(r=0.888); GABRG2(r=0.878); GLRB(r=0.877) |
|  | E | NECTIN3(r=0.897); PLPPR4(r=0.893); SYNE1(r=0.875) |
| postsynaptic organization | C | PAFAH1B1(r=0.893); GLRB(r=0.89); CAPRIN1(r=0.883) |
|  | D | DNM1L(r=0.915); PAFAH1B1(r=0.914); PRNP(r=0.905) |
|  | E | CDKL5(r=0.819); LRRTM1(r=0.793); ZNF804A(r=0.779) |
| presynaptic membrane | C | NPTN(r=0.879); SNAP91(r=0.864); SCN2A(r=0.849) |
|  | D | DNM1L(r=0.915); CNTN1(r=0.888); NRXN1(r=0.864) |
|  | E | CASK(r=0.89); GRIN2A(r=0.843); SLC1A6(r=0.836) |
| synaptic plasticity | C | NPTN(r=0.879); PPP3CB(r=0.874); RAB11A(r=0.86) |
|  | D | PRNP(r=0.905); FGF14(r=0.879); MAPK1(r=0.87) |
|  | E | GRIN2A(r=0.843); CALB1(r=0.837); RIMS1(r=0.817) |
|  | I | CPLX2(r=0.752); PRRT2(r=0.712); BRSK1(r=0.707) |
| immune response | F | LRRC25 (r=0.876); EGFR (r=0.860); CC2D2B (r=0.8524) |
|  | K | ISYNA1 (r=0.921); CYP2W1 (r=0.915); PRRC2C (r=0.906) |
|  | L | FCGR1B(r=0.765); FCGR1B(r=0.725); C1QC(r=0.719) |
|  | R | APOL2(r=0.783); REG3A(r=0.744); TFR2(r=0.721) |
| mitochondrion | A | TP53BP2(r=0.925); PON2(r=0.897); GJA1(r=0.894) |
|  | C | VDAC1(r=0.928); SUCLA2(r=0.927); TMEM14A(r=0.925) |
|  | D | YWHAZ(r=0.927); IDH3A(r=0.923); ABHD10(r=0.922) |
|  | H | OGDHL(r=0.892); RAB11FIP5(r=0.83); PC(r=0.818) |
|  | M | ISCU(r=0.916); BCL2L2(r=0.893); ATPAF1(r=0.892) |
|  | I | MRPL41(r=0.665); MRPS26(r=0.654); MRPL52(r=0.651) |
| DNA damage | C | COPS5(r=0.889); COPS8(r=0.851); COPS8(r=0.835) |
|  | D | UBE2N(r=0.912); PAK1(r=0.892); UBE2D3(r=0.887) |
|  | G | NEK4(r=0.862); ZBTB38(r=0.858); SMC3(r=0.853) |
|  | N | CUL4B(r=0.858); RAD21(r=0.805); BRCC3(r=0.755) |
|  | I | BRSK1(r=0.707); NOP53(r=0.538); TAOK2(r=0.312) |
| autophagy | C | BECN1(r=0.908); VMP1(r=0.906); RAB1A(r=0.895) |
|  | D | MAP1LC3B(r=0.905); RAB1A(r=0.887); ATG5(r=0.872) |
|  | I | MAP1S(r=0.758); WDR45(r=0.756); ATG9A(r=0.712) |
|  | N | NBR1(r=0.768); MFN1(r=0.664); ATG12(r=0.661) |
| lysosome | B | LAMP2(r=0.925); NPC1(r=0.917); LAMP2(r=0.914) |
|  | D | ARL8B(r=0.91); ATP6AP2(r=0.889); SNX2(r=0.882) |
|  | L | DTX3L(r=0.723); VAMP8(r=0.673); HCK(r=0.658) |
| proteasome complex | C | PSMC6(r=0.895); UBQLN1(r=0.884); PSMA1(r=0.881) |
|  | D | PSMA1(r=0.867); PSMD10(r=0.856); UCHL5(r=0.823) |
|  | L | PSMB8(r=0.602); PSMB9(r=0.52); NA(r=NA) |
|  | H | BAG6(r=0.501); BAG6(r=0.46); NA(r=NA) |
| stem cell maintenance | A | SOX2(r=0.866); FOXO1(r=0.823); SOX2(r=0.735) |
|  | C | PSMC6(r=0.895); PSMA1(r=0.881); PSMA3(r=0.872) |
|  | L | PSMB8(r=0.602); PSMB9(r=0.52); NA(r=NA) |
|  | I | CNOT3(r=0.589); NA(r=NA); NA(r=NA) |
|  | N | SMC3(r=0.661); RIF1(r=0.629); MED7(r=0.611) |
| viral process | B | NPC1(r=0.917); NCAM1(r=0.892); MOG(r=0.869) |
|  | C | VDAC1(r=0.928); SUMO1(r=0.912); BECN1(r=0.908) |
|  | D | G3BP2(r=0.945); RAB6A(r=0.9); VDAC1(r=0.899) |
|  | G | TANK(r=0.848); MORC3(r=0.831); IPO7(r=0.804) |
|  | L | C1QA(r=0.717); CD93(r=0.703); HCK(r=0.658) |
|  | N | CREB1(r=0.803); KPNA5(r=0.791); ZMYND11(r=0.762) |
|  | K | TRIM31(r=0.734); TRIM8(r=0.663); NA(r=NA) |
|  | M | IFIT1(r=0.813); ADAR(r=0.766); VAPB(r=0.745) |


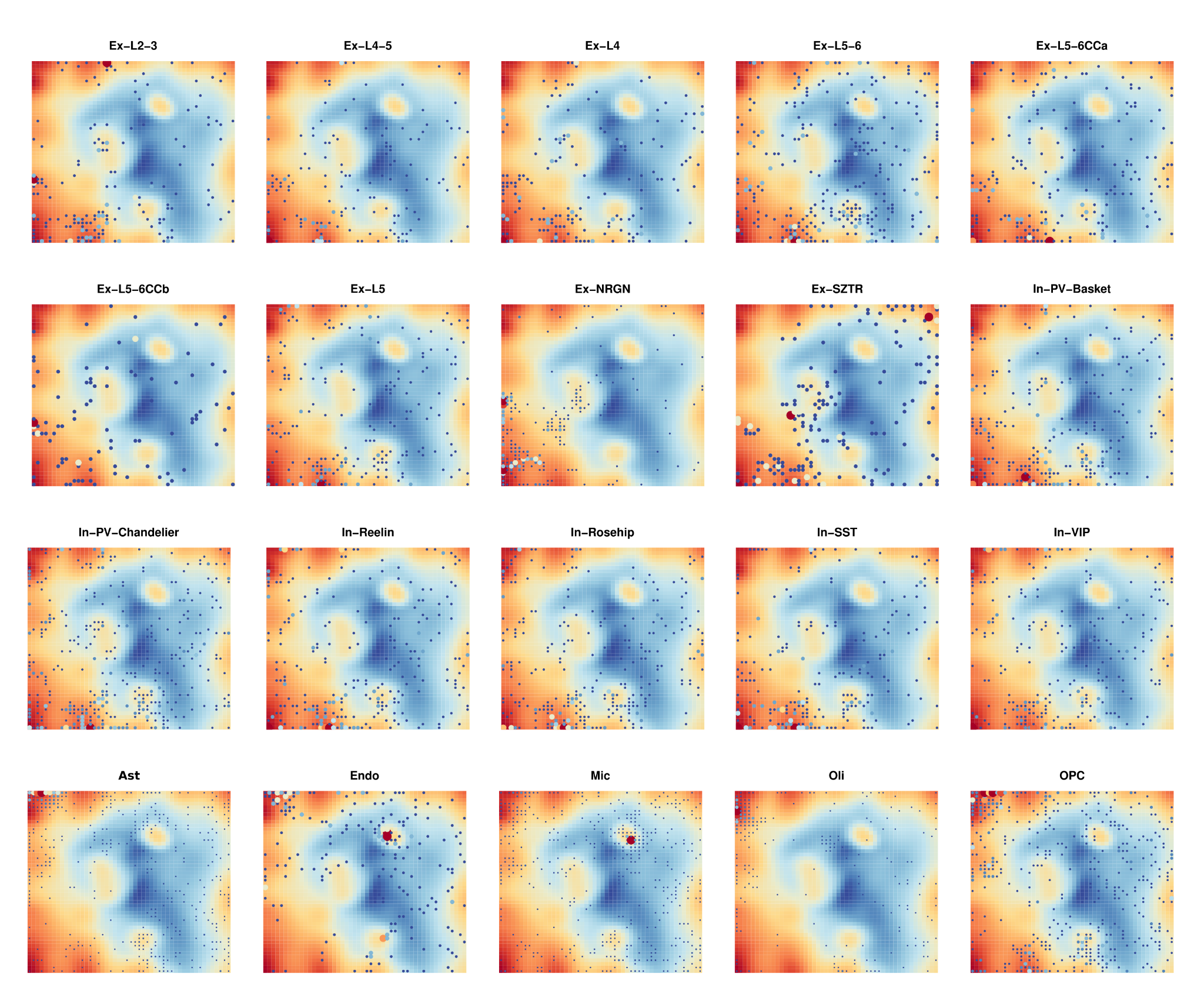


**Figure S4.** Projection of brain cell population gene signatures on the global SOM landscape. Dots represent genes projected to the corresponding meta-gene on the SOM landscape. The size of the dots represents the number of genes from a given meta-gene. “Oli” - oligodendrocytes, “Mic” - microglial cells, “Ast” - astrocytes, “Endo” - endothelial cells, “OPC” - oligodendrocyte precursor cells, “In-Reelin” – reelin–expressing interneurons; “In-PV-Chandelier” – parvalbumin (PV)-expressing chandelier interneurons; “In-PV-Basket” parvalbumin (PV)-expressing basket interneurons; “In-SST” – somatostatin (SST)-expressing interneurons; “In-Rosehip” – Rosehip interneurons; “In-VIP” – vasoactive intestinal polypeptide (VIP)–expressing interneurons; “Ex-L2-3” – Layer 2/3 excitatory neurons; “Ex-L4” – Layer 4 excitatory neurons; “Ex-L4-5” – Layer 4/5 excitatory neurons; “Ex-L5” – Layer 5 excitatory neurons; “Ex-L5-6” – Layer 5/6 cortico-fugal projection excitatory neurons; “Ex-L5/6-CCa” – deep-layer 5/6 cortico-cortical projection excitatory neurons enriched for dopamine receptor signaling pathway and “Ex-L5/6-CCb” – deep-layer 5/6 cortico-cortical projection excitatory neurons enriched for glutamate signaling; “Ex-NRGN” – neurogranin (NRGN)-expressing excitatory neurons; “Ex-SZRT” – excitatory neurons of “schizophrenia transcriptional resilience” type/state (it is preferentially found in schizophrenia individuals whose transcriptional profiles are non-schizophrenia-like).

**
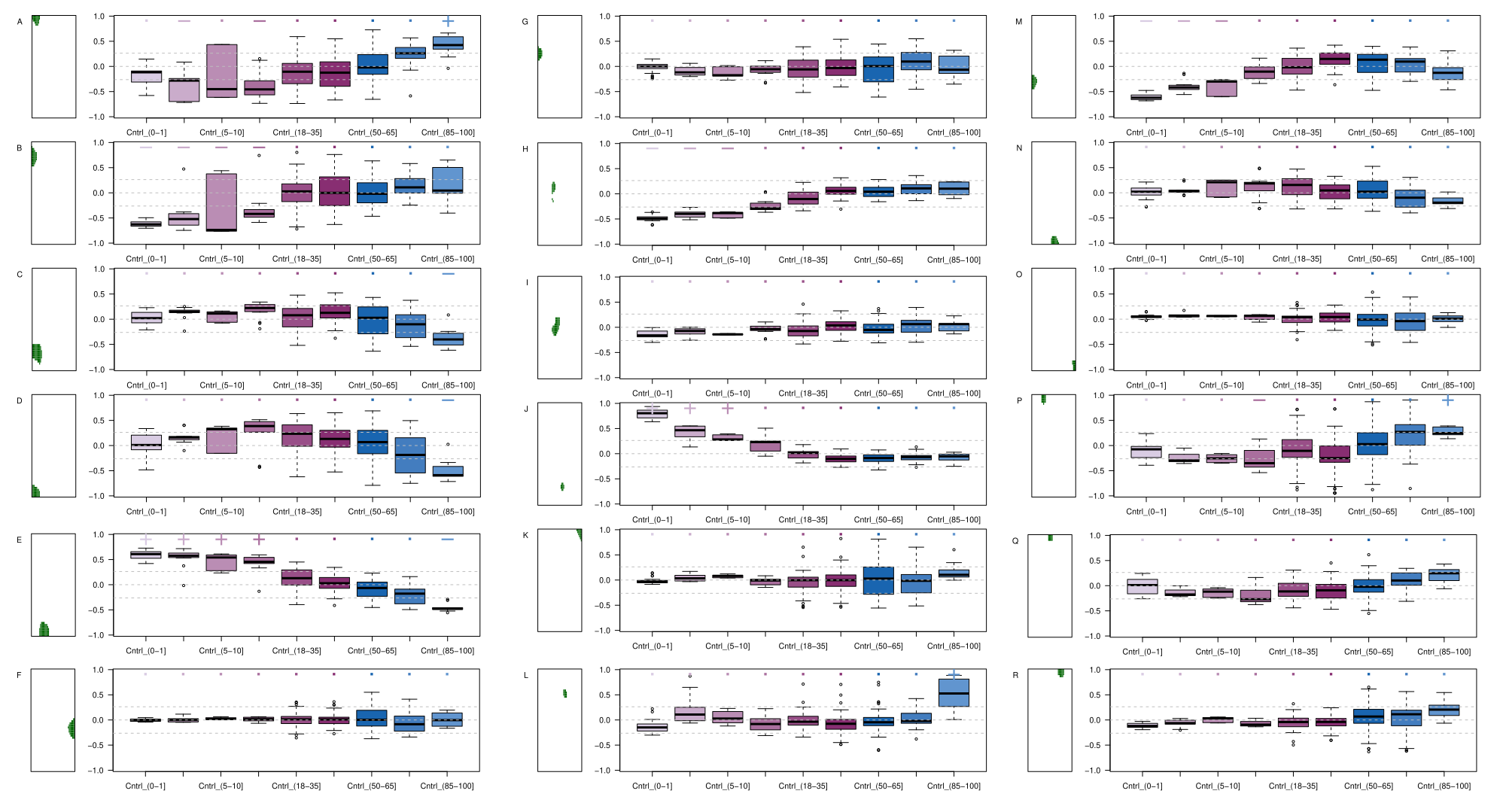
**

**Figure S5.** Age-related changes in spot expression in control samples.

**
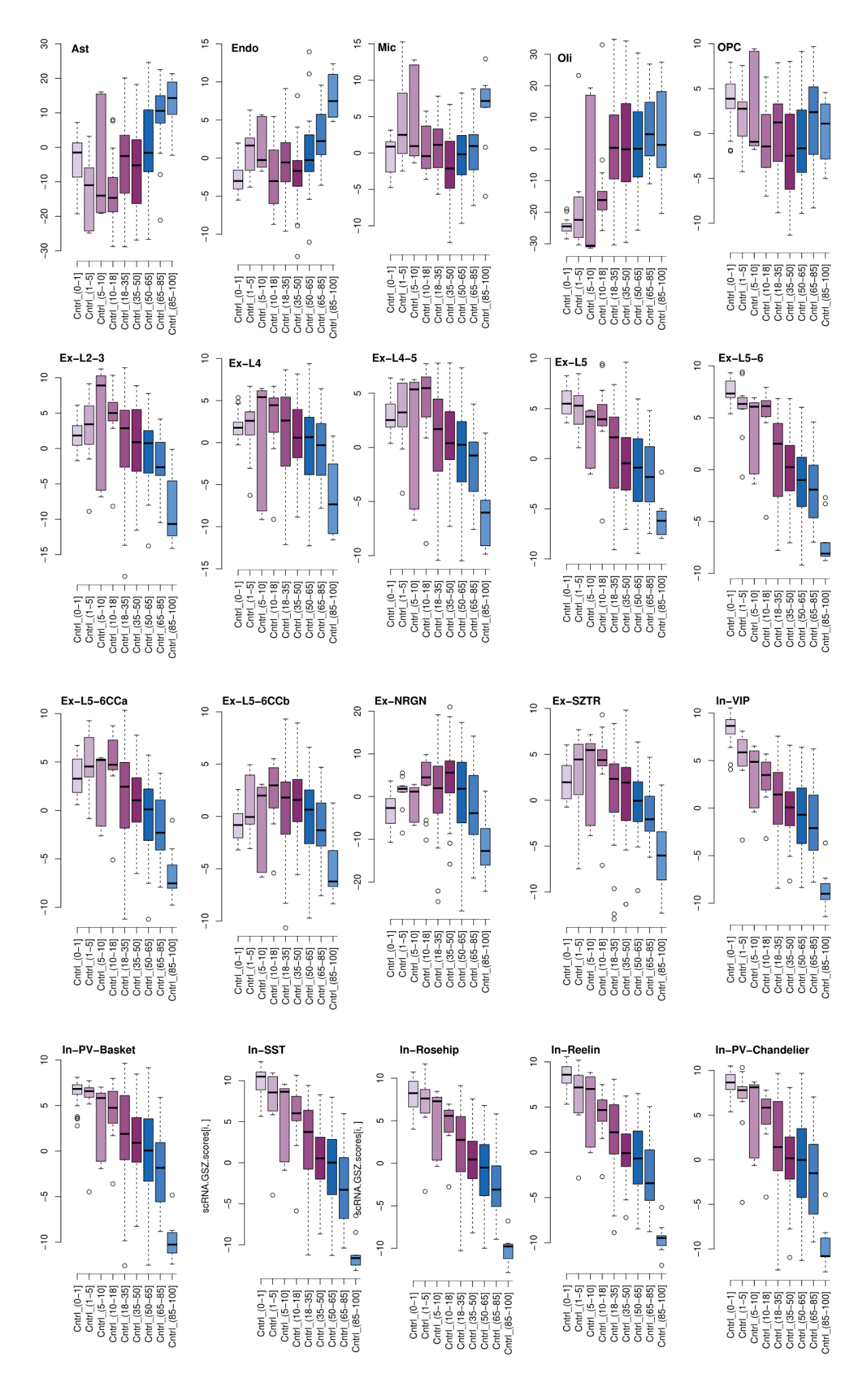
**

**Figure S6.** Age-dependent changes in GSZ-scores of brain cell population gene signatures. “Oli” - oligodendrocytes, “Mic” - microglial cells, “Ast” - astrocytes, “Endo” - endothelial cells, “OPC” - oligodendrocyte precursor cells, “In-Reelin” – reelin–expressing interneurons; “In-PV-Chandelier” – parvalbumin (PV)-expressing chandelier interneurons; “In-PV-Basket” parvalbumin (PV)-expressing basket interneurons; “In-SST” – somatostatin (SST)-expressing interneurons; “In-Rosehip” – Rosehip interneurons; “In-VIP” – vasoactive intestinal polypeptide (VIP)–expressing interneurons; “Ex-L2-3” – Layer 2/3 excitatory neurons; “Ex-L4” – Layer 4 excitatory neurons; “Ex-L4-5” – Layer 4/5 excitatory neurons; “Ex-L5” – Layer 5 excitatory neurons; “Ex-L5-6” – Layer 5/6 cortico-fugal projection excitatory neurons; “Ex-L5/6-CCa” – deep-layer 5/6 cortico-cortical projection excitatory neurons enriched for dopamine receptor signaling pathway and “Ex-L5/6-CCb” – deep-layer 5/6 cortico-cortical projection excitatory neurons enriched for glutamate signaling; “Ex-NRGN” – neurogranin (NRGN)-expressing excitatory neurons; “Ex-SZRT” – excitatory neurons of “schizophrenia transcriptional resilience” type/state (it is preferentially found in schizophrenia individuals whose transcriptional profiles are non-schizophrenia-like).

**Table S2.** Spot-level transcriptome perturbation time points in schizophrenia

| Spot | log-likelihood ratio | MAP (years) * | ptl5 | ptl95 |
| --- | --- | --- | --- | --- |
| A | 7.45 | 0.11 | 0.11 | 34.36 |
| C | 12.45 | 0.11 | 0.11 | 11.85 |
| D | 17.96 | 0.11 | 0.11 | 7.94 |
| E | 16.96 | 0.11 | 0.11 | 7.94 |
| K | 1.48 | 0.11 | 0.11 | 47.09 |
| Q | 14.87 | 0.11 | 0.11 | 6.96 |
| R | 2.23 | 0.11 | 0.11 | 89.17 |
| N | 14.29 | 9.89 | 0.11 | 15.77 |
| M | 5.32 | 13.81 | 1.09 | 25.55 |
| L | 8.52 | 28.49 | 3.04 | 50.02 |
| I | 1.38 | 62.74 | 2.06 | 76.45 |

* MAP - maximum posterior probable age, ptl5 - 5 percentile of the posterior distribution, ptl95 - 95 percentile of the posterior distribution

**Table S3.** Spot-level transcriptome perturbation time points in bipolar disorder

| Spots | log-likelihood ratio | MAP (years) * | ptl5 | ptl95 |
| --- | --- | --- | --- | --- |
| A | 11.14 | 0.11 | 0.11 | 10.54 |
| O | 1.03 | 0.11 | 2.00 | 87.36 |
| G | 1.43 | 14.33 | 1.06 | 80.72 |
| P | 5.90 | 25.71 | 2.00 | 33.30 |
| Q | 2.71 | 34.25 | 2.95 | 36.15 |
| C | 2.15 | 35.20 | 2.00 | 71.24 |
| L | 1.70 | 55.12 | 8.64 | 56.06 |

* MAP - maximum posterior probable age, ptl5 - 5 percentile of the posterior distribution, ptl95 - 95 percentile of the posterior distribution

**Table S4.** Spot-level transcriptome perturbation time points in major depressive disorder

| Spots | log-likelihood ratio | MAP (years) * | ptl5 | ptl95 |
| --- | --- | --- | --- | --- |
| F | 3.37 | 0.11 | 0.11 | 30.46 |
| G | 2.06 | 0.11 | 1.06 | 52.27 |
| H | 2.50 | 0.11 | 0.11 | 88.31 |
| I | 1.62 | 0.11 | 1.06 | 90.21 |
| K | 2.74 | 0.11 | 1.06 | 45.63 |
| M | 4.14 | 0.11 | 0.11 | 52.27 |
| C | 3.24 | 13.38 | 1.06 | 51.32 |
| P | 4.56 | 25.71 | 2.00 | 31.40 |
| L | 1.52 | 28.55 | 3.90 | 70.29 |

* MAP - maximum posterior probable age, ptl5 - 5th percentile of the posterior distribution, ptl95 - 95th percentile of the posterior distribution

**Table S5.** Transcriptome perturbation time points for metagenes (spots) in psychiatric diseases.

| Spot ID | Maximum Posterior Probable Age (years) | | | Associated processes |
| --- | --- | --- | --- | --- |
|  | SCZ | BD | MDD |  |
| A | 0.11 | 0.11 |  | action potential, axon guidance, calcium signaling, mitochondrion, stem cell maintenance |
| C | 0.11 | 35.2 | 13.4 | dendrite development, postsynaptic density, postsynaptic organization, presynaptic membrane, mitochondrion, DNA damage, proteasome complex, stem cell maintenance, viral process |
| D | 0.11 |  |  | action potential, calcium signaling, dendrite development, GABA signaling, glutamate signaling, neurodevelopment, postsynaptic density, postsynaptic organization, presynaptic membrane, synaptic plasticity, mitochondrion, DNA damage, autophagy, lysosome, viral process |
| E | 0.11 |  |  | action potential, axon guidance, calcium signaling, dendrite development, GABA signaling, migration, neurodevelopment, postsynaptic density, postsynaptic organization, presynaptic membrane, synaptic plasticity |
| F |  |  | 0.11 | immune response |
| G |  | 14.3 | 0.11 | DNA damage, viral process |
| H |  |  | 0.11 | mitochondrion, proteasome complex |
| I | 62.7 |  | 0.11 | postsynaptic density, mitochondrion, DNA damage, autophagy, stem cell maintenance |
| K | 0.11 |  | 0.11 | immune response, viral process |
| L | 28.5 | 55.1 | 28.6 | immune response, lysosome, proteasome complex, stem cell maintenance, viral process |
| M | 13.8 |  | 0.11 | action potential, mitochondrion, viral process |
| N | 9.9 |  |  | DNA damage, autophagy, stem cell maintenance, viral process |
| O |  | 0.11 |  | regulation of T cells, cholesterol transport |
| P |  | 25.7 | 25.7 | mRNA splicing, transcription |
| Q | 0.11 | 34.3 |  | translation, centrosome |
| R | 0.11 |  |  | immune response, acute phase response, signal transduction |


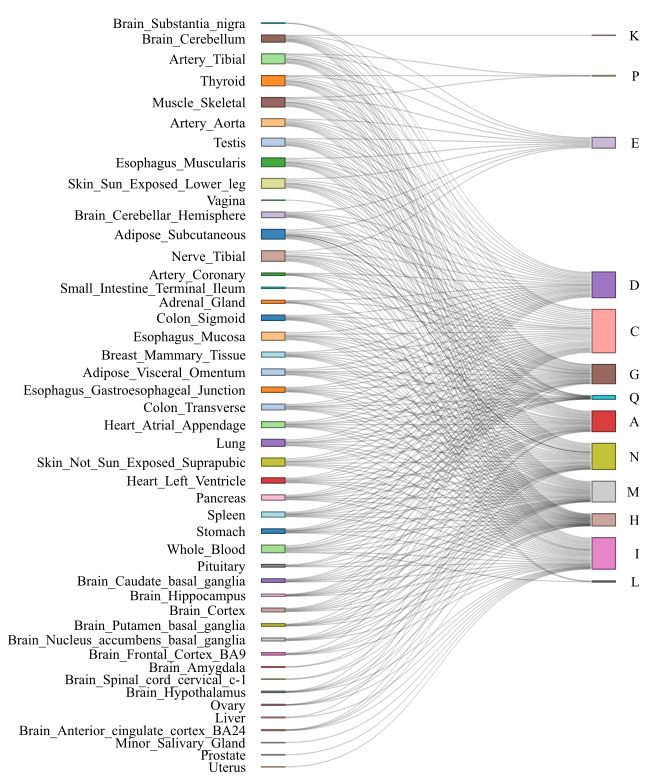


**Figure S7.** Tissue eQTL enrichment in time-perturbed spots for all mental disorders. Links represent significant enrichment (adjusted Fisher Exact p-value < 0.05) of tissue-specific eQTLs with spots.

**Table S6.** Association of disease terms related gene sets with genes from eQTL enriched spots.

| Database | Term | Overlap | Nominal p-value | Adjusted p-value | Odds Ratio |
| --- | --- | --- | --- | --- | --- |
| DisGeNET | Schizophrenia | 791/1923 | 4.87E-33 | 2.32E-29 | 1.82 |
| DisGeNET | Other specified types of schizophrenia, unspecified | 30/53 | 2.49E-05 | 0.0007 | 3.19 |
| DisGeNET | SCHIZOPHRENIA 1 (disorder) | 31/58 | 8.21E-05 | 0.0018 | 2.82 |
| GWAS Catalog 2019 | Response to antipsychotic treatment in schizophrenia | 7/7 | 0.00017 | 0.083 | 99351 |
| DisGeNET | Bipolar Disorder | 190/837 | 7.66E-08 | 4.69E-06 | 1.59 |
| DisGeNET | Major Depressive Disorder | 152/513 | 0.00043 | 0.022 | 1.40 |
| DisGeNET | Unipolar Depression | 149/517 | 0.0017 | 0.049 | 1.35 |
| DisGeNET | Depression, Bipolar | 24/61 | 0.0035 | 0.08 | 2.15 |
| UK Biobank GWAS v1 | Longest period of depression 4609 raw | 217/791 | 0.0029 | 0.047 | 1.26 |
